# IMPROV FOR CARE: TEACHING CAREGIVERS IMPROVISATION IMPROVES MOOD AND SENSE OF BURDEN

**DOI:** 10.1093/geroni/igz038.3133

**Published:** 2019-11-08

**Authors:** Ruth Almen, Jessica Z Kirkland Caldwell

**Affiliations:** 1 Cleveland Clinic Nevada Lou Ruvo Center for Brain Health, Las Vegas, Nevada, United States; 2 Cleveland Clinic Lou Ruvo Center for Brain Health, Las Vegas, Nevada, United States

## Abstract

There is a growing need for novel intervention for caregivers of family members with dementia. Improv for Care is a six-week program designed to teach improvisation (“improv”) skills to caregivers to practice flexible communication, build social support, and process the demands of caregiving through humor and play. This study aimed to examine changes in caregiver depression (Beck Depression Inventory-II), perception of burden (Zarit Burden Interview), qualitative experiences related to caregiving, and their cared-for person’s neuropsychiatric symptoms (Neuropsychiatric Inventory Questionnaire). Fifteen caregivers completed questionnaires before and after the Improv for Care program. Wilcoxon signed rank tests for related samples revealed significant declines in both caregivers’ depressive symptoms, Z = -2.64, p = .008, and sense of burden, Z = -2.16, p = .031, after completing the program. Caregivers reported that their loved ones’ neuropsychiatric symptoms increased during the course of the intervention, Z = -2.10, p = .036, though associated distress did not also increase, Z = -1.12, p = .265. Of the 15 caregivers, 12 completed follow up questionnaires three months after course completion, which showed that their post-intervention reduction in depressive symptoms remained stable, Z = -1.02, p = .306. The Improv for Care program shows promise as an intervention for caregivers to improve stress, mood, and coping skills.

